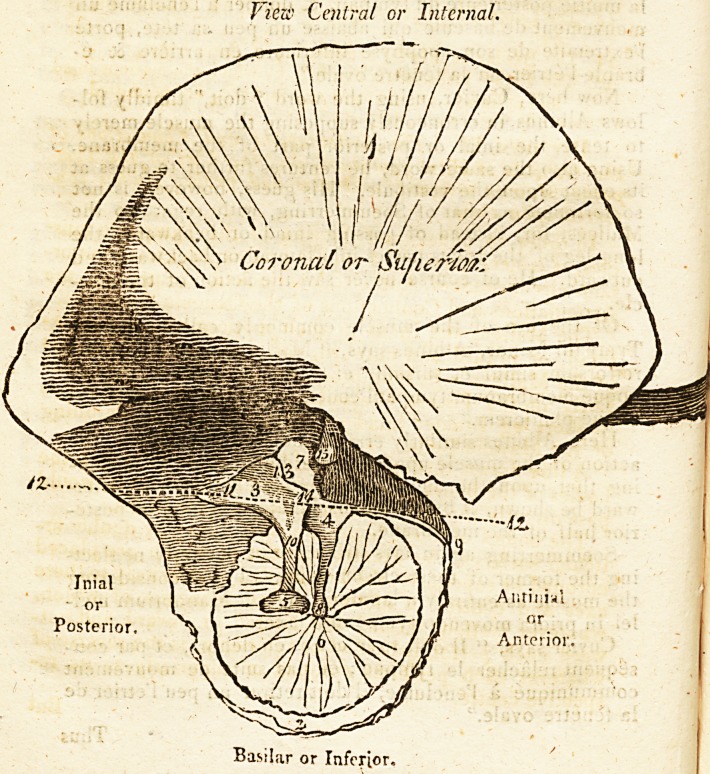# Theory of Phonics, Hearing, &c.

**Published:** 1808-05

**Authors:** Alexander Walker

**Affiliations:** Lecturer on Physiology at Edinburgh


### Medical and Phyfical Journal.

VOL. XIX.] MAY, 1808. [NO. 111.

*Printed for R. PHILLIPS, by W. Thorne, Red Lion Court, Fleet Street, London*

### THEORY OF PHONICS, HEARING, &c.

### PHYSIOLOGICAL DISSERTATION ON THE FUNCTIONS OF THE OSSICULA AUDITUS, AND OF THE TYMPANIC MUSCLES IN PARTICULAR, AND ON THOSE OF THE EAR IN GENERAL;

*Proving that the articulations and connexions of the Ossicula Auditus have not hitherto been understood; that the functions of the Tensor Tympani have hitherto been only partially known; that the functions of the muscles improperly termed Laxatores Tympani have hitherto been still less understood, and more especially that the very curious, beautiful, and important functions of the Stapideus have hitherto been entirely unknown; explaining also all the articulations and connexions of the Ossicula, assigning the real uses of the Tympanic Muscles, and conferring upon them new and proper names; as well as giving an outline of an original System of the Physiology of the Ear, and involving an original Theory of Phonics.* BY ALEXANDER WALKER, ESQ. LECTURER ON PHYSIOLOGY AT EDINBURGH.

DIVISION I. *The Anatomy and more especially the Physiology of the Ear, though much cultivated, by no means understood.*

THAT this is the case I am convinced by the number of discoveries which a minute investigation into the structure and functions of this organ has enabled me to make. I have observed, that the Membrana Mucosa of the fœtus is, in reality, a membranous bag, or consists of two layers of which the circular edges are connected, and which, examined after death, contains a quantity of fluid in which flakes of a whitish substance float.—I have observed, that the Ossicula Auditus are, instead of four, as has been supposed, only three in number; the os lenticulare being merely a process of the incus, connected to it by bony substance, and having a cellular structure joining that of the last mentioned bone. In the publication of this fact, however, although I had often mentioned it, and had put one preparation illustrating it into the hands of Dr. Monro, and another into those of Dr. Barclay, and although a friend sometime afterward published the fact in his thesis, yet I believe I am, upon the whole, anticipated by Professor Blumenbach of Gottingen. I have also observed that the muscle commonly called Stapideus, arises not by one, but two origins, viz. one from the cavity of the Pyramis Tympani, and another from the inside of that part of the Fallopian Aquæduct which is contiguous to, and communicates with it.

Now as these discoveries were made respecting the very first parts of the ear to which I turned my attention, and as there are many other desiderata respecting it, (several of which in the following pages I have answered,) I am led to conclude, that numerous facts of the same kind must have escaped the eyes of Cassebohm, Scarpa, Monro, and Soemmerring. Nor are these facts, though minute, unimportant, because, without such a knowledge of the structure of an organ initself the most minute, the functions of these parts can never be explained. And, indeed, ignorance of such minutiæ is no inconsiderable cause of the total ignorance which has hitherto existed respecting the physiology of the ear; I therefore conceive myself to be borne out in the assertion, that the anatomy of the ear is by no means understood.

As to the disgraceful state of its physiology, it will be evident to every one who recollects our being gravely assured that the want of the external ear, of the membrâna tympani, and of the ossicula auditus make no difference upon the sense, or who knows, that we have hitherto been ignorant of the still more important and beautiful functions which I am about to describe.

### Division II. *Their great Importance to the Discovery of the Intellectual Functions in general.*

Before we can possibly understand the functions of the nerves, we must understand those of the brain on which these chiefly depend, and before it is possible to understand those of the brain, we must understand those of the organs of sense, whence impressions are transmitted to it, and upon which all its functions consequently depend.

In order, therefore, to discover the functions of all the intellectual organs, the strict physiological method; the only only method calculated to lead to success, is first to discover the functions of the organs of sense.

Of all these, I choose to begin with the ear, because, though it has hitherto been least understood, it presents, as I shall have an opportunity of showing, that which, if I might use the term, I should call a real analysis of a sensitive organ.

Division III. *The Nature and Particular Objects of the Present Enquiry.*

The present enquiry is particularly directed to the Ossicula Auditus and the Muscles of the Middle Ear, because, though these last are little known, yet, in consequence of their situation, and the powers which, from their attachment to very movable and delicately constructed bones, they possess, they equally influence the external, middle, and internal ears, and illustrate their physiology, and also on account of the peculiar interest and beauty which the subject possesses.

It will be seen, that all of them, in a peculiar manner, tense or relax the Membrana Tympani, the Membrana Fenestræ Ovalis, the Membranes, the Fluid, and, consequently the Nerves of the Labyrinth, and through them also the Membrana Fenestræ Rotundæ in a manner hitherto unknown.

I hope it will appear, that considerations of such extensive influence in an investigation into the functions of the intellectual organs, and which assigns the uses, hitherto unknown, and confers new and appropriate names upon four pairs of the human muscles, are amply sufficient for the mind to rest upon for a period. Besides, I shall in the Sixth Division of the paper, which assigns the uses of these muscles and confers upon them new and proper names, endeavour to exhibit such an original specimen of minute anatomy, accurate reasoning upon structure and strict physiological induction concerning the most complex and least understood organ in the body, as I shall not fear to have placed in comparison with that of any other anatomist or physiologist. And this I mention by no means with a view of enhancing my own weak abilities, but for the purpose of showing, by a comparison of which I do not dread the result, the utility of the mode of investigation which I have adopted. If, however, there should be any doubt concerning this method and its result, I have only to say, "The comparison is easy."

Previously, however, to explaining the uses of these muscles, it will be proper to consider their attachments and situation together with their uses, as described by the most celebrated anatomists and physiologists, accompanying this with critical remarks, pointing out the errors they have committed.

Division IV. *Of the Attachments and Situation of the Tensor Tympani, of the Muscles improperly termed Laxatores Tympani, and of the Stapideus, according to Albinus, Soemmerring, and Cuvier, with Critical Observations.*

The muscle usually called Tensor Tympani, is thus described by Albinus, "Similis Externo Mallei Tensor Tympani, sed major. A superiore hic parte tubæ Eustachianæ, qua nempe tuba illa calvariæ basem spectat, assimilisque est naturæ cartilaginæ, circa ossis multiformis foramen admittens vasa ad duram matrem, tenuissimo principio, abscidit: unde procedens, primo modice crassior fit, latiorque; dein rursus gracilior, donec in tendinem vertatur. Procedit retrorsum ad tympanum, dein per id ipsum, modice simul et extrorsum et sursum declinans. Mox autem ab ortu recipitur in semicanalem, qui ejus causa partim supra tubæ osseam partem, partim in superiore et eadem priore parte tympani, e regione membranæ tympani, formatus est; atque in eo vagina quadam tendinosa, quæ canalem perficit, retinetur, ne elabatur. Ad cujus canalis finem cum tendo ejus pervenit, exit ex eo, flectitque se circum vaginam, decurrens membranam tympani versus, simulque modice retrorsum : seseque ad postremum mallei manubrio inserit, infra processum ejus gracillimum ; et ei quidem manubrii parti, quæ spectat fundum tympani, oppositum membranæ."

Of the same muscle Soemmerring says, "Principio tendineo a parte superiore cartilaginis tubæ acusticæ juxta partem sphenoidalem ossis spheno occipitalis, vel ab ipso sphenoidali parte ortus, et canali pyramidis semiosseo et semiligamentoso inclusus, fusiformis. Tendinem e canali emersum, et quasi circumtrochleam ductum ad latus mallei, qua cavum tympani spectat, mittit."

Cuvier says, "L'interne, qui vient de la partie cartilagineuse de la trompe, marche dans un demi-canal pratiqué dans le rocher sur la partie osseuse de la trompe; peu après son entrée dans la caisse, il rencontre une éminence située en avant de la fenêtre ovale, et nommée bec-de-cuiller. Il contourne son tendon sur une traverse de cette eminence; et le dirigeant en dehors, l'insère au manche du marteau, a sa face interne, et sous son apophyse grêle."

Of

Of these descriptions, that of Albinus is so eminently superior, that I cannot help deprecating the conduct of the other two anatomists, who, succeeding him, could presume to give such loose descriptions; and I blame it less in the comparative than in the human anatomist, to whose genius anatomy and physiology are so deeply indebted. To the mere description of this muscle by the first of these great men, I myself can add nothing.

Albinus thus describes the muscle usually called Stapideus, "Stapideus quoque ventrem et tendinem habet. Venter hæret in cavernula ossis petrosi, quæ in tympano ante inferiorem partem aquæductus Fallopii est: ex eaque oritur, et per eandem oblique in priora adscendit. At tendo ejus ex ejusdem cavernulæ rotundo ore exit, et quamprimum exiit, flectit se per tympanum in priora, rectaque petit posteriorem partem capituli stapidis.

Of the same muscle Soemmerring says, "Fusiformi et flexo ventre in cavernula ossea cavi tympani incluso, teres tantum tendo, ex osse emersus, prominet, qui parti posteriori capituli stapedis inseritur."—Nonnunquam tendo ejus latus manubrii posterius sequitur, donec ei prope apicem inseritur."

Cuvier says, "Le muscle de l'etrier est placé dans un creux d'une éminence située en arrière de la fenêtre ovale près du bord posterieur de la caisse, et qu'on a nommee éminence pyramidale; son tendon en sort pour se porter directment à la branche posterieure de l'etrier."

Of these descriptions of the Stapideus, that of Albinus is also generally preferable; but Soemmerring has noticed what he deems a variety in its insertion; viz. that it is sometimes fixed to the posterior crus of the stapes. Cuvier seems to consider this as its usual insertion, and my observations have frequently accorded with his.—None of these anatomists, however, nor any other anatomist, has hitherto correctly described the origin of this muscle, which is, in reality, double. Its short head, which alone they have observed, arises, as they have described, but its long head, which they have not noticed, arises from within the Fallopian Aquæduct, where it communicates with and crosses laterad,* or external to the cavity of the Pyramis Vestibuli, within which it joins the shorter head.—In

 passing

* Throughout this paper, I use the terms of the New Nomenclature, because accurate description, especially of the minute kind, cannot be conveyed in any other terms. The common terms, however, also accompany them.

passing through the tympanum to be inserted, the course also of the muscle differs from that which anatomists have hitherto assigned to it, as it does not pass directly antiniad or forward, but antinio-centrad or forward and inward.

The muscle usually called Laxator Tympani Major is thus described by Albinus, "Externo Mallei, quamvis tantulo, et venter, et tendo, sicut multis maximorum. Venter oblongus est, et ab acuto initio primo crassior, mox gracilescit, atque in tendinem, se multo longiorem, abit. Oritur abexteriore parte acuti processus, quem ultimus ossis multiformis angulus, inter os squamosum et petrosum intersertus, exigit; procedit secundum finitimum commissurum ossis squamosi et petrosi, atque adeo oblique in latus retrorsum modiceque sursum: rimam deinde quæ ad finem modo dictæ commissuræ inter eadem illa ossa relicta, tendine suo intrat, posteaque se retrorsum flectens, pergit per eum extremi osseæ partis illius, quæ porum acusticum continet, sinum, per quam malleus processum suum longissimum eundemque gracillimum porrigit; totique se processus illius longitudini affigit."

Of the same muscle, Soemmerring says, "Tumidulo ventre a processu spinoso ossis spheno occipitalis ortus, tendinem longum per rimam, cavum glenoidale inter et meatum acusticum, ad processum mallei longum mittit."

Cuvier says, L'externe marche parallèlment au précédent, mais plus en dehors, et s'insère à l'apophyse grêle du marteau, qui est elle-même logée dans un petit canal pratiqué au-dessus du bord supérieur du cadre du tympan. Ce muscle est si foible qu'on a peine à s'assurer de sa vraie nature.

Of this muscle as usual, the description of Albinus excels that of the other anatomists. The concluding remark of Cuvier, that "Ce muscle est si foible, &c." would induce one to think that he had never seen the muscle, and that, as he makes no such remark respecting the succeeding one, the proper object of such a remark, he had in mistake applied it to this. Cuvier, as will afterward appear, is too often conducted by guess.

Albinus thus describes the muscle usually called Laxator Tympani Minor: "Inter mallei musculos minimus Laxator Tympani; qui a nobis, ni quid forte in re tam subtili difficilique hallucinati sumus, adhuc inventus est oriri ibi ubi membrana tympani adhæret poro acustico; et quidem juxta illius pori partem eandum et superiorem, et nonnihil posteriorum. Sensim autem gracilior, manubrio se mallei juxta radicem processus brevioris affigit, extremo gracillimo; limo; toto decursu descendens introrsum, et modice in priora."

Of the same muscle, Soemmerring says, " A superiori et posteriori margine meatus acustici cui membrana tympani adhæret, ortus, sensimque extenuatus, et ad interiora et priora descendens manubrio mallei juxta processum breviorum inseritur."

Cuvier says, " Le laxateur vient de la voûte du méat externe, près le tympan, passe par l'échancrure du cadre de cellui-ci, et s'insère à la petite saillie oblique du col du marteau."

Respecting this muscle, I need only say, that the description of Albinus still excels that of the other two anatomists, but he errs in saying that it passes *introrsum* to be inserted. Its course is actually the reverse, and it was by guessing its action from this erroneous supposition of its course, that Albinus thought it, in any measure, a Laxator: I have only to add respecting it, that the muscle lays in the basilo-inial, or inferior and posterior edge of a very fine duplicature of the tympanic periosteum. The above alteration, and this addition being made, the description of Albinus is correct.

DIVISION V. *Of the Uses of these Muscles according to the same Anatomists.*

It is upon this subject, rather than the former, that I decidedly differ from these celebrated men; for although Albinus excels the others no less in the uses which he assigns to the tympanic muscles, than in the descriptions which he gives of them; yet, upon this, the most difficult part of myology, even he is throughout defective or erroneous. The attempt, however, which Soemmerring, and more especially Cuvier makes to supply his defects, leaves them still behind him. These anatomists proceeded by guess, and upon this subject, uniformly terminated in error.

With regard to the use of the muscle commonly called Tensor Tympani, Albinus says, " Hic musculus ob singularem suum decursum aptus est ad mallei manubrium à poro acustico retrahendum ad oppositum tympani partem, et modice eodem tempore in priora; quo fit, ut cum malleum sequatur membrana tympani, eam intro trahere valeat, ac tendere, cavamque efficere à parte pori acustici."

Now Albinus greatly errs in saying that this muscle draws the handle of the malleus *in priora*, and this error he was led into by the blamable practice of guessing at uses from structure, or in other words, he reasoned from one part of the structure, viz. the oblique direction of the muscle,

 without without noticing the oblique direction of the articular surfaces which completely counteracts its operation. He also neglects the action of the muscle on the labyrinth.

Of the use of the same muscle, Soemmerring says, "Mallei ope tympanum in caverne tympani retrahit, eoque modo tantum tendit, ut cavum a priore parte efficiat. Dubio procul incudis ope stapidem a priore parte in labyrinthi vestibulum urget."

In the last of these sentences, Soemmerring evidently guessed at the use of the muscle. The words "procul dubio" by no means imply his having seen it, but, in reality, suppose the reverse. The guess, however, is an excellent one, and will be confirmed in the sequel.

Of this guess Cuvier avails himself by merely translating the words "procul dubio urget" into "doit pousser," which is only continuing the guess, thus: "Il tire le marteau entier en dedans, et tend le membrane du tympan; et par le mouvement que le marteau communique à l'enclume, la jambe supérieure de celle-ci, restant fixée, l'autre doit décrire un arc de dehors en dedans, et pousses l'etrier dans la fenêtre ovale."

Of the use of the muscle usually called Stapideus, Albinus says, "Hoc igitur capitulum (nempe stapedis) ad os cavernulæ attrahit, quo ita movet stapidem, ut pars posterior basis ejus in vestibulum auris introeat, prior ab eodem recedat."

Now Albinus is, like all other anatomists and physiologists, totally ignorant of the very important use of this muscle, with regard to the membrana tympani, nor does he even understand its real action on the base of the Stapes; for it presses no part of it against the vestibulum, but in consequence of its oblique insertion and the additional obliquity of the fenestra ovalis, its action pulls the bone almost directly from that cavity.

Soemmerring, following Albinus, plunges deeper in the same error, by assigning to the muscle an use totally opposite to that which it actually serves; thus: "Partem basis stapidis posteriorem in vestibulum urget; musculum tympani tensorem igitur ab altero latere adjuvat, ut eo directius stapes in fenestram ovalem urgeatur."

Cuvier also says of it, "Tire (l'etrier) en arrière, en soulevant un peu sa partie anterieure."

Thus the use of this muscle, equal in importance to the tensor, has hitherto been entirely unknown, and affords a clear proof that anatomists, instead of experimenting on the action of these muscles, have hitherto only guessed at them.

With

With regard to the use of the muscle commonly called Laxator Tympani Major, Albinus says, "Malleum in priora trahit; modiceque parum acusticum versus; ex quo malleum sequens membrana tympani planior fit, et modice laxior; inæqualiter, maxime laxata illa parte, quæ à priori parte mallei."

Now, here Albinus not only entirely neglects its action on the vestibulum, but mistakes that upon the membrana tympani; for, as will afterwards be shown, it by no means laxates the antinial or anterior half of this membrane, as that anatomist supposed.

Soemmerring not only errs in neglecting the former of these circumstances, but in considering the muscle as entirely a laxator; thus, "Malleum in priora versus meatum acusticum movet, eoque modo tympanum laxat."

Cuvier says, "Il doit tirer le marteau en avant; tendre la moitié postérieure du tympan, & donner à l'enclume un mouvement de bascule qui abaisse un peu sa tête, porte l'extremite de son apophyse inférieure en arrière & ébranle l'étrier sur la fenêtre ovale."

Now here, Cuvier, using the word "doit," timidly follows Albinus in erroneously supposing the muscle merely to tense the inial or posterior part of the membrane. Using also the same word, he ventures further to guess at its effect upon the vestibule. His guess, however, is not so fortunate as that of Soemmerring, with regard to the Malleus, for, instead of passing iniad or backward, the long leg of the Incus passes inio-laterad or backward and outward. He of course never saw the action of the muscle.

Of the use of the muscle commonly called Laxator Tympani Minor, Albinus says, "Mallei manubrium trahit retrorsum simul et sursum, et parum acusticum versus; eoque membranum tympani eodem versus trahit, laxat, facitque planiorem."

Here Albinus similarly errs by not only neglecting the action of the muscle upon the vestibulum, but by mistaking that upon the membrana tympani, for, as will afterward be shown, it by no means laxates the inial or posterior half of the membrane.

Soemmerring again errs still more in not only neglecting the former of these circumstances, but in considering the muscle as entirely a laxator; thus, "Manubrium mallei in priora movendo tympanum laxat."

Cuvier says, "Il doit tirer cet os en dehors, et par conséquent relâcher le tympan; et pas suite de mouvement communiqué à l'enclume, il doit retirer un peu l'etrier de la fenêtre ovale."

Thus

Thus, in assigning the use of this muscle, Cuvier again using the word "doit," follows Soemmerring rather than Albinus in considering it an entire laxator, and so having no direct antagonist to his last mentioned muscle. Using also the same word, he ventures to guess at its action upon the vestibule; but his guess is again indistinct and abortive. He gives no clear or accurate account of the action of the muscle. Of course he never actually saw it.

In concluding this Division, the Author need only remark, that the result of these criticisms, as they prove the failure even of the great Albinus, ought for ever to prevent Anatomists from guessing at the uses of parts when absolute experiment is within their reach.

### SKETCH AND EXPLANATION,

TO ILLUSTRATE THE SUBSEQUENT DIVISION.

*View Central or Internal.*

Central — toward the Spectator, or Interior.
Lateral — from the Spectator, or Exterior.

1. The Squamous portion of the Temporal Bone.
2. The Osseous ring of the Membrana Tympani.
3. The Incus.
4. The Malleus.
5. The Base of the Stapes removed from the Fenestra Ovalis.
6. The Membrana Tympani.
7. The Head of the Malleus.
8. The Handle of the Malleus
9. The Long process of the Malleus.
10. The Long leg of the Incus.
11. The Short leg of the Incus.
12. Line passing through the Axes and Centres of Motion mesiad and laterad, or inward and outward of the Malleus and Incus.
13. The Coronal or superior articulation of the Malleus and Incus.
14. The Basilar or inferior articulation of these bones.
15. The Pivot, hitherto unobserved, by which the motions iniad and antiniad, or backward and forward, are permitted.

Division VI. *The Articulations and Connections of the Ossicula Auditus explained, and the Uses of the Tympanic Muscles assigned, and new and proper Names conferred upon them.*

In order to understand the uses of the Tympanic muscles, it is necessary that I should first explain some important circumstances, hitherto overlooked, respecting the Articulations and Connections of the Ossicula Auditus.

The Malleus and Incus in their motions mesiad and laterad, or inward and outward, roll upon Axes. The axis of the Malleus is its long process situated antiniad or anteriorly in the Fissura Glasseri; and that of the Incus is its short leg situated iniad or posteriorly in the beginning of the Mastoid Cells.

A line drawn through the middle of these axes and the bodies of the bones which are placed between them, would pass directly through the centres of these motions.

The bodies of these bones are also joined by double articular surfaces, the greater portions of which are placed coronad or superiorly, the less basilad or inferiorly to these axes and centres of motion; in consequence of which, whatever motions mesiad and laterad or inward and outward each performs basilad or inferiorly, is the reverse of whatever each performs coronad or superiorly to its respective centre.

But

But these axes can only regulate the motions mesiad and laterad or inward and outward, and it is necessary that similar ones should regulate the motions iniad and antiniad or backward and forward. For this purpose, the point of the axis of the Incus is retained in its situation by a strong ligament, inserted into a depression on its mesial or inner side, and by another passing over its antinial or anterior side, and becomes a new centre of motion, while the greater portion of it, admitting free and conspicuous motion coronad and basilad or upward and downward, forms an axis for motions iniad and antiniad or backward and forward, as well as for motions mesiad and laterad or inward and outward. But the axis of the Malleus being movable iniad and antiniad or backward and forward, cannot form an axis for motions in these directions. Besides its axis, therefore, the Malleus possesses a pivot, which projects coronad or superiorly to the bony ring of the membrana tympani and antinio-basilad or anteriorily and inferiorily to its own head, which is adapted to the pivot by a depression so slight as not to interfere with the motions mesiad and laterad or inward and outward. And, in consequence of this pivot and the above mentioned axis of the Incus, the motions of that bone and the Malleus iniad and antiniad or backward and forward, are permitted.

Now the articular surfaces of these bones are not of a simple, but of a complex kind; those of the Incus receiving those of the Malleus; in consequence of which reception, when in the chief motions of the bone, i. s. those which take place mesiad and laterad or inward and outward, that portion of the Malleus which is basilar or inferior to its centre of motion is drawn mesiad or inward, it must press its basilar or inferior articular surface against the basilar or inferior articular surface of the Incus, and so force mesiad or inward all the portion of that bone which is basilar or inferior to its centre of motion; when, on the contrary, the long leg of the Incus is drawn laterad or outward, the contrary motions must take place; and, in general, whatever may be the motion mesiad and laterad or inward and outward of the parts of the bones basilar or inferior to the centre of motion, which now appears to be common, the motions of the parts coronal or superior to the centre of motion must be the reverse.

Thus far I have, for the sake of easy illustration, supposed the motions of these two bones exactly to coincide. But did they exactly coincide, articular surfaces would not have have existed, and osseous union would have best contributed to these coincident movements; neither could their movements, which are incoincident, i. s. those which take place iniad and antiniad or backward and forward, have been performed.

They do not, however, coincide exactly, because their articular surfaces are oblique and twisted. For, although the Malleus is situated antiniad or forward, and the Incus iniad or backward, their articular surfaces have neither of these aspects, which could only have suited coincident motions iniad and antiniad or backward and forward, and incoincedent motions mesiad and laterad or inward and outward, veiy nearly the reverse of those which were necessary; but, while the articular surfaces of the Incus receive those of the Malleus, the basilar or inferior articular surface of the Incus has the latero-corona-antinial or external, superior and anterior aspect, and that of the Malleus the opposite mesio-basilo-inial or internal, inferior and posterior aspect; and the coronal or superior articular surfaces, being less twisted and complex, that of the Incus has only the antinio-mesial or anterior and internal aspect, and that of the Malleus the opposite inio-lateral or posterior and external aspect. Besides, their capsular ligament is sufficiently loose to allow these oblique and twisted surfaces to glide upon each other.

Now, while this obliquity of articular surfaces, this looseness of the capsular ligament and gliding motion, together with the separation of the handle of the Malleus and long leg of the Incus are indispensibly necessary to permit the incoincident motions iniad and antiniad or backward and forward, the coincident motions mesiad and laterad or inward and outward, are not, in the slightest degree, deranged by the obliquity of the articular surfaces, being counteracted by the oblique direction of the tympanic muscles, which most beautifully modifies their motions, so that the same general effect is produced as if the articular surfaces had been directly, instead of obliquely, opposed to each other's motion, and the muscles directly instead of obliquely, inserted.

I may now observe, in order to explain the mutual action of the bones, that as the basilar or inferior articular surface of the Incus has, in a great degree, the lateral or external aspect, and the corresponding surface of the Malleus, in an equal degree, the mesial or internal, while the coronal or superior articular surface of the Incus has, in a certain degree, the mesial or internal aspect, and the corresponding surface of the Malleus, in an equal degree, the the lateral or external — aspects directly opposed to each other, motions somewhat coincident mesiad and laterad or inward and outward must be easily performed; but still, as oblique surfaces would considerably oppose the coincidence of these motions, in order that their effect might be, in a certain degree, obviated, the muscles destined to produce these motions mesiad and laterad or inward and outward, are, as I have mentioned, obliquely inserted.

In consequence of this structure, when any force pulls the handle of the Malleus mesiad and slightly antiniad or inward and slightly forward, which is nearly in a mean direction to the obliquity of the articular surfaces, these surfaces are almost equally opposed to each other, and the portions of the bones basilar or inferior to their centre of motion pass together mesiad; but as, after all, such a force is more at right angles to the basilar or inferior articular surfaces than to the coronal, these surfaces are more opposed, become in a greater measure the centre of motion; the coronal or superior surfaces slightly diverge; and consequently such a force causes the handle of the Malleus and the long leg of the Incus slightly to converge in passing mesiad. And this, as will be seen, is the case of the Tensor Tympani.

In consequence of the same structure, when any force pulls the long leg of the Incus inio-laterad or backward and outward, which is precisely at right angles to the coronal or superior surfaces, these surfaces of the bones remain opposed to each other, and, in a certain degree, fixed, while the basilar or inferior articular surfaces and portions of the bones at once diverge and pass laterad. And this, as will be seen, is the case of the muscle usually called Stapideus.

I may now further observe, that as the basilar articular surface of the Incus has, in some degree, the antinial or anterior aspect, and the corresponding surface of the Malleus, in an equal degree, the inial or posterior, while the coronal or superior articular surface of the Incus has, in a greater degree, the antinial or anterior aspect, and the corresponding surface of the Malleus, in an equal degree, the inial or posterior — aspects directly opposed to each other, motions iniad and antiniad may be easily performed. But as, in these motions, the axis of the Malleus is changed from its long process to its pivot, the former, in consequence of its bent direction, passing easily in the new direction assumed by the bone, and the latter being placed antinio-basilar or forward and downward to the head head of the Malleus, and so permitting the corresponding part of the bone to rotate upon it, and its head to descend or ascend upon the body of the Incus, while the short leg of that bone, becoming an axis for motions in a different direction, viz. basilad and coronad or downward and upward, the motions iniad and antiniad or backward and forward of the two bones cannot be coincident, but, when the body of the Incus is made by the head of the Malleus to descend, the long leg of the one and the handle of the other must diverge, and, when the body of the Incus is made by the head of the Malleus to ascend, the long leg of the one and the handle of the other must converge.

In consequence of this structure, when any force pulls the Malleus antinio-laterad or forward and outward, its head, in this case rotating upon the pivot antinial or anterior to it and descending upon the two articular surfaces, presses the body of the Incus basilad, or downward, and, in consequence of the descent of the body of that bone its long leg passes iniad or backward, while, at the same time, it is carried with the Malleus slightly laterad or outward. And this, as will be seen, is the case of the muscle usually called Laxator Tympani Major.

In consequence of the same structure, when any force pulls the handle of the Malleus inio-laterad or backward and outward, its head, in this case rotating upon the pivot antinial or anterior to it, and ascending between the two articular surfaces, presses the body of the Incus coronad or upward, and, in consequence of the ascent of the body of that bone, its long leg passes antinial, while, at the same time, it is carried with the Malleus slightly laterad or outward. And this, as will be seen, is the case of the muscle usually called Laxator Tympani Minor.

It remains for me now to explain why it is necessary that, in those motions which are most coincident, and which carry the handle of the Malleus and long leg of the Incus mesiad and laterad, or inward and outward, these parts of the bones should diverge or converge instead of being entirely coincident in their motion. Now, this curious motion is absolutely necessary on account of the oblique positions of the membranes which they tense or relax; for the lateral or external surface of the membranæ tympani, instead of having the direct lateral aspect, has one somewhat antinial or anterior, and the membrana Fenesceræ Ovalis, instead of the direct lateral aspect, has one somewhat inial or posterior; in consequence of which, in order directly to tense these, it is necessary that the handle handle of the Malleus should pass latero-antinial, or outward and forward, and the long leg of the Incus latero-iniad or outward and backward—in other words, that they should diverge.

Respecting the third bone of the ear, I have to observe, that the cause of its posterior leg being longest, is to give such obliquity to its base as is necessary to adapt it to the oblique position of the Membrana Fenestræ Ovalis, and such apposite obliquity to its head as is necessary to enable it to meet the handle of the Malleus.

It is also the oblique direction—the inial or posterior aspect of the Membrana Fenestræ Ovalis, that permits the Stapideus, notwithstanding its direction iniad or backwark, to draw the Stapes as most directly from the Fenestra; and it is partly from overlooking this direction of the membrane, that anatomists and physiologists have so long remained ignorant of the use of the muscle.

Having premised these indispensible observations, I may now explain the uses of each of the Tympanic muscles, and confer upon them such names as their uses demand.

Lest, however, any one should imagine, that the uses ascribed to these muscles by me, have been merely deduced from the preceding facts respecting the articulations and connections of the ossicula auditus, and from the reasoning which I have entered into respecting them, but not actually seen, I must observe, that the most important and original of all these uses, viz. that of the Stapideus, was demonstrated to my friend, Dr. Barclay, in a preparation made by me in his own presence, and, that that gentleman was entirely satisfied with the demonstration, is evident from his having, in his invaluable work, On the Muscular Motions of the Human Body, mentioned the communication. Not only this, however, but all the motions of the other muscles which I have mentioned, have been demonstrated to my friend Dr. Sanders, and even the actions of some of the muscles upon the fluid of the labyrinth, were exhibited to Dr. Barclay by the rising and falling of fluid in the divided superior semicircular canal of a bone which had been previously preserved in spirit.

Of the Muscle usually called Tensor Tympani.—The use of the muscle commonly called Tensor Tympani, with regard to the Membrana Tympani is, by means of its attachment to the Malleus, to pnll it mesiad or inward and so render its lateral or external surface concave. —The use of this muscle has been long understood, and is the the only one of the uses of any of the tympanic muscles which has been known.

With regard to the labyrinth, the use of this muscle is, while the Malleus and Incus rest upon their axes, by pulling centrad or inward the basilar or inferior articular surface of the Malleus against the basilar or inferior articular surface of the Incus, to force the depending or long leg of the latter mesiad or inward, and consequently to push the Stapes, attached to it, against the Membrana Fenestræ Ovalis, and so diminish the cavity and render tense all the membranes of the labyrinth.—This last use of the muscle was suspected by Soemmerring, and after him by Cuvier, who, however, had evidently not seen the fact, and does not appear to be absolutely certain about it.

In consequence of these uses, this muscle (if its name shall express its use) must be called Auris Membranarum Tensor, or the Tensor of all the Membranes of the Ear.

Of the Muscle usually called Stapideus.—The use of the muscle commonly called Stapideus, with regard to the Membrana Tympani is, while the Incus and Malleus rest upon their axes, by pulling laterad or outward the head of the Stapes against the depending or long leg of the Incus, to press also laterad the basilar or inferior articular surface of the Incus against the basilar or inferior articular surface of the Malleus, and consequently to force the handle of that bone laterad or outward, and so relax the Membrana Tympani.

With regard to the labyrinth, the use of that muscle is, by pulling the Stapes antinio-laterad or forward and outward, to relax the Membrana Fenestræ Ovalis, and consequently to enlarge the cavity and relax all the membranes of the labyrinth.

In consequence of these uses, this muscle must be called Auris Membranarum Laxator, or the Laxator of all the Membranes of the Ear.

Of the Muscle usually called Laxator Tympani Major.—With regard to the Membrana Tympani, the use of the muscle commonly called Laxator Tympani Major is, by pulling the handle of the Malleus antinio-corono-mesiad or forward, upward and inward, over the edge of the bony ring of the membrani tympani, to tense it in these directions.

With regard to the labyrinth, the use of this muscle is, by causing the Malleus to rotate upon its pivot, and its head to descend upon the body of the Incus, to push the long leg of that bone and the Stapes in the same direction, and similarly to incline the Membrana Fenestræ Ovalis.

In consequence of these uses, this muscle must be called Auris Membanarum Antinio-mesiad Tendens, or the Tensor of all the Membranes of the Ear inward and forward.

Of the Muscle usually called Laxator Tympani Minor.—With regard to the Membrana Tympani the use of the muscle commonly called Laxator Tympani Minor, is, by pulling the handle of the Malleus inio-corono-mesiad or backward, upward and inward, to tense it in these directions.

With regard to the labyrinth, the use of this muscle is, by causing the Malleus to rotate upon its pivot, and its head to ascend upon the body of the Incus, to push the long leg of that bone and the Stapes in the same direction, and similarly to incline the Membrana Fenestræ Ovalis.

In consequence of these uses, this muscle must be called Auris Membranarum Inio-mesiad Tendens, or the Tensor of all the Membranes of the Ear backward and inward.

Division VII. *Rapid View of an Original Theory of Phonics and of Musical Sounds; in Illustration of the subsequent Theory of Hearing.*

Motion.—The impulse of bodies against each other is the cause of all the variety of sounds, and the simpler the structure of a sonorous body is, the simpler is the sound it emits when impelled. Although no substance perhaps emits a simple sound, yet I am of opinion, for reasons which I shall subsequently assign, that a sonorous plate emits the simplest; and that, contrary to the common doctrine, the sound emitted by a musical string is much more complex. This I shall subsequently explain. As to variety of sounds, it is commonly asserted in a vague manner, that the human voice produces the greatest number of them; but this assertion is by no means correct, for, though the human voice produces the greatest number of what I should term tones or sounds of different quality, it does not produce the greatest number of pitches or sounds of different elevation.

All sounding bodies evidently vibrate; the vibration of the larger ones is even sensible to the eye, but very distinct to the touch. These vibrations also take place in various directions, with various force and frequency, and in various combinations. The numerous varieties of sounds therefore therefore arise from the directions of the vibrations (this curious fact I shall subsequently prove), the number of the vibrations, the frequency of the vibrations, and the combinations of the vibrations. Hence arise the quality, the height, the force, and the complexity of sounds. Short and slender strings or pipes produce high, acute, or sharp sounds; long and wide ones the reverse; large vibrations produce strong or loud sounds, small ones the reverse; a single complex body or various simple ones, not in unison, produce complex or mixed sounds.—An irregular or complex body produces complex sounds, in consequence of its various parts performing their vibrations in different times. The number of the vibrations of other sonorous bodies are generally ascertained by comparing them with those of musical strings. The continuance of these vibrations is according to the greater or less elasticity and the thickness or thinness of the sounding body.

Air is the only substance which seems constantly to exist between sounding bodies and the ear. Hence we have reason to conclude, that it is the vehicle of sonorous vibration. The vibrations of the air are even evidenced by those of the dust, smoke, and light particles which float in it. Sounds or rather vibrations, however, are conveyed by liquids as well as by fluids. Sounds are even louder in the dense air of vallies than in the rarer air of mountains. Solid bodies convey them still more intensely; this is exemplified by the different intenseness of the sound produced by a watch, first placed on the further end of a long metallic rod, the near end of which approaches the ear, and then suspended at the same distance in the air. Solid bodies then transmit them most rapidly. This better transmission of sounds by solid bodies is illustrated by the practice among savage nations, of applying the ear to the ground in order to distinguish the approach of their enemies.

It is generally observed, that it is questionable whether sound is transmitted through a vacuum; but there can be very little doubt on this point, when the analogy upon the subject is considered, viz. that solids transmit it best, and the rarer bodies worst; besides it is evident, that that which possesses no medium of transmission cannot be transmitted. The experiments performed upon this subject in the imperfect vaccua are unworthy of notice. Sound, however, is enfeebled by passing from one medium to another, and its intensity diminishes according to the distance. The exact ratio, however, of its diminution is not known.

Very strong sounds communicate motion to bodies of every kind. Even less sounds, however, are not merely communicated to the external air, but very curiously to all such bodies as, if struck, would emit a sound similar to the original one, or, in other words, would produce similar vibrations. This is well illustrated by the string of a violin, which is tuned in unison with another over which a bow is drawn, sounding or rather vibrating at the same instant, while other strings not in unison are not at all moved. This is a consequence of the vibrations of the air being performed in times similar to those of the sounding body, and of the motions which its particular tension permits, being in the second string exactly similar to those which the first string and the air are performing. The case of a pendulum, of which the motion is impeded by impulses either more or less frequent than its own oscillations is a beautiful illustration of this fact. Further, if, of two strings tuned in unison, one is capable of performing them while the other performs one vibration, then the latter will divide itself into three vibrating points, and there will be two points at rest, as may easily be evidenced by placing small bits of paper on the last mentioned string.

The propagation of sound is much slower than that of light, but it is at an uniform rate, which is about 1142 feet per second, and the undulation of water is to the motion of air, according to Hales, as 1 to 865. The velocity of sound is also the same whether high or low. Nevertheless, whatever increases the elasticity of air seems to increase the velocity and intensity of sound. And, in fluids of a determinate elasticity, whatever increases their density diminishes the velocity of sound. It is this exact degree of the velocity of sound that enables us to measure distances, wherever colour is seen and sound at the same time proceeds. The velocity of sound, however, is slightly altered by wind. But it is in consequence of this fact, that, by adding the velocity of the wind to, or subtracting it from that of sound, we are enabled to measure the velocity of the wind. It is, however, far inferior to that of sound. *This last fact, in my opinion, clearly shows that two sorts of motion take place in the air.*

The vibratory motion of a sounding body is communicated all round, precisely as the undulations of water, and it is wrong to remark, as any exception to this analogy, that particular directions may be given to sound, for that can only be effected by obstructing it in other directions which may as easily be effected in liquids. The crossing of of aerial, may also be explained by the crossing of aquatic undulations. All this, however, refers only to one of the species of motion that takes place in the air; for, as I above observed, the greater velocity of sound than that of wind proves, in my opinion, that there are two, and that, *while the communication of sound is modified by the former, its very existence seems to depend upon that which I am now to explain.*

*It appears to me, that it is upon a more minute direction of the aerial particles that the various modifications of sound depend, and that, upon the degree or extent of this direction, their degrees of strength depend. But, as sound, instead of being capable of transmission through vaccua, seems to me evidently to depend on the formation of minute vaccua within bodies, or the media which transmit it, and upon the collapse of the particles forming the sides of these vaccua, it is evident that we ought not merely to consider the direction of the vibrations, but rather the form and direction of the intervals upon which the sound still more immediately depends.*

*Now it appears to me, that the direction of these vibrations, or rather of these intervals, must uniformly be of a circular kind. In order to illustrate this, I shall choose, as an example, the intervals produced by the vibration of a sonorous metallic plate. Suppose this plate to be held at one edge, as the leaf of a book is attached to the back, (for all sounding bodies must have one point more fixed than others) and that its loose or free edge is struck laterally, it is evident that as one edge is fixed, the other must move upon it as an axis — must move, as it were, from side to side, and that however small its vibrations, they must form small segments of a circle, the radius of which is equal to the width of the plate. This appears to me to be incontestibly evident. Hence then the minute intervals which it leaves at every point of these segments must be circularly arranged, or have a circular direction. Nor does all this take place merely in a sonorous plate, for upon this principle must all other sonorous bodies vibrate. We shall presently see how beautifully applicable these facts are to the illustration of the phenomena of hearing.*

In order more completely to illustrate this Theory of Vibration, I may here explain, upon the same principle, the action of a vibrating string. Now it will appear, that the action of a string is, as I before asserted in opposition to the common opinion, much more complex than that of a plate; so much so, that I am utterly amazed natural philosophers should ever for a moment have thought the

 reverse.

reverse. First then it is evident, that the string being fixed at two ends, and vibrating in the centre, forms, from that very circumstance, on each side of its central point, two series of segments, the convexities of which are turned toward each other, and, in that single point of view, is doubly more complex than the plate. But even this is effected by the mere edge of the string, or the central line of one of its sides; and a little consideration will I think show, that the sides of the string which are separated by this edge must, as they have different aspects, throw off the particles of air in different directions from each other and from the edge between them. Hence the complexity is multiplied, and will be still increased by its vibrating, when strongly struck, even in different planes. Thus, in vibrating strings the small ones produce slender sounds by a slender aperture between the two extremes of vibration; the large ones, the reverse. The advancement then of a string in its first, and retrocession in its second vibration, must occasion successive aeriform undulations.

We know, that elastic bodies of an oblong form, when rubbed longitudinally, emit sounds, and that their longitudinal are higher than their lateral vibrations. This last appears to me to arise from their particles being more opposed to each other in that direction, and therefore forming, in their vibrations, smaller segments, precisely as greater tension operates in strings. And that the height of the longitudinal vibrations is, nevertheless, inversely as the length of the sonorous body, is to be accounted for from this, that, in very long bodies rubbed longitudinally, the lateral vibrations must also take place. From this fact of the existence of longitudinal vibrations, it is very properly concluded, that the extension and contraction of a string in its lateral vibrations must produce longitudinal ones; but when it is asserted that this will also be a cause of the string producing two different sounds, the conclusion is erroneous; for the lateral and the longitudinal vibrations are in this case coincident, and the one will only modify the effect of the other — in other words, a composition of forces is produced, and the aerial particles move in their diagonal.

It is also known, that musical strings form different curves, accordingly as they are struck with different substances and in different directions. A vibrating string may also be made to move irregularly in different planes; and sometimes a string seems to perform one general and several subordinate vibrations.

It

It is upon this principle, that I would account for the circumstance of performers on the violin or harp producing tones slightly different from the same string, by affecting it at different places, or even in the same place. In affecting it at different places, they will increase the particular curves which that part of the string assumes, and render more audible the particular tones which these curves produce; in affecting it at the same place, by the different directions of the impulse, they may cause it to move in different planes, and to produce slightly different sounds. I am convinced that it is, in reality, a *practical* knowledge of these facts that chiefly enables one performer to excel another.

The various forms assumed by light bodies upon the surface of a sonorous plate, when the bow of a violin is drawn across its edge, as proposed in the experiment of Chladni, may appear, at first sight, to militate against the present theory, that sonorous vibrations, or rather intervals, uniformly possess a circular form. But I must observe, that whenever these vibration figures assume any other than a circular form, it is in consequence of the performer causing, by the manner of holding the plate, such a number of vibrating centres, that the forms proceeding from the one are modified by those proceeding from another.

I may conclude this part of the subject by merely observing, that sounds are capable of being reflected, and hence arises the echo, which is best produced by the most solid bodies, and is heard soonest by those nearest the reflecting substance, while by those who are equally distant from it and from the surrounding body only one sound is heard. The angle of the reflection of sound is equal to its angle of incidence. Hence echoes are best heard in the direction corresponding to it. Smooth projecting solids give the best echo; on a plain none is heard. Convex bodies are bad reflectors of sound, in consequence of their dispersing it; flat ones are better; concave ones, especially when the sounding body is in the focus, are best.

From the Theory of Phonics in general, I may now proceed to sketch the Theory of Musical Sounds, which results from it.

From what I have already said, the nature of distinct or simple sounds must now be evident. In Music, Melody is a succession of simple sounds, and Harmony is a succession of complex or coexistent sounds, two of which are called Consonant or Dissonant, and a greater number

Accordant or Discordant, according to their pleasant or unpleasant effect. The art of music, therefore, is that which expresses sounds in such arrangement as to produce *expressive* Melody and Harmony.

The number of vibrations performed by musical strings, in other respects equal, are inversely as their length; if chords differ in thickness only, the number of their vibrations are inversely as their diameter; if chords differ in tension only, the number of their vibrations are as the square roots of the stretching weights. Hence similar chords may give different and different the same pitches; hence also the number of vibrations which a stretched string can perform may be easily ascertained where its tension, length, and diameter are known. Thus it is that the most powerful and the feeblest instrument, though they differ in strength, may be adjusted so as to produce their vibrations in the same times, and consequently to produce the same pitch, which if strong, in music, is called forte, if weak piano.

Tones in music are improperly used to express the intervals between the pitches; they ought to be used to express sounds of different quality or of different kinds. In this sense, tones are as numerous as the simple vowels which the voice produces; they are indeed the same, or exactly correspond with them. Hence, while the human voice produces numerous tones or vowels, musical instruments, singly considered, produce only one. They are also inferior to the voice in this, that they are totally incapable of producing those sounds called articulate. This, however, they in some measure compensate for, in producing a great variety of pitches.

The same chord performs both its large and small vibrations in similar times; of course its sounds, though weaker, are of one pitch. Now, the particular pitches which are chosen in consequence of their pleasant effect, are, when arranged in a particular order, denominated the Scale of Music. As in this scale, each eighth pitch is of double the elevation or depression of that from which it is reckoned, the whole scale is supposed to consist of octaves. Musical pitches, however, do not, like those of the human voice, ascend in a regular series, but leave intervals of different magnitude between them, and Major Tone, Minor Tone, and Semitone are the terms which express the magnitude of these intervals between the pitches. There exists however, a method of supplying these intervals when large, by the interposition of other intermediate mediate pitches. In some instruments, all these pitches are fixed; in others, they must be determined by the judgment of the performer, who, from the facility or difficulty with which he executes this, is said to have a good or a bad intonation. In the singing of birds, it is remarked, that though the separate tones and pitches are clear and beautiful, they are not arranged at musical intervals, they have consequently little expression, and produce neither melody nor harmony of a pleasant kind.

There is a great defect, however, arising from the choice and arrangement of musical pitches, by which when D, for instance, is the key note, A, which was a perfect sixth to C, when it was the key note, is an imperfect fifth to D. Nor can the interposition of one string remedy this, because though perfect fifth to D, it would be an imperfect 4th when E, or improper 3d when F was key note. And if, in each case, another string was introduced, there would be an endless multiplicity. In order to get rid of this defect, which is called the wolf in music, it is necessary to tune the particular string in an intermediate way, so that the imperfection is divided; A, for instance, being rendered an imperfect sixth to C, and an imperfect fifth to D. Even this division, however, is made unequally in fixed instruments, in order to answer the particular keys in which most pieces are written.

When two instruments are in unison, they are said to be of the same pitch.—The most perfect consonance is between any note and its octave, then its fifth, then its third sharp. This consonance or dissonance of sounds seems to arise from the coincidence of the vibrations. A succession of thirds, however, is more pleasant than of fifths, and a discord in certain accords is agreeable. A perfect accord is that which arises from any note, its third sharp, fifth, and its octave. The rest are imperfect, yet may be introduced with effect.

There exist in music two very remarkable phenomena; first, that if a large string of an instrument be sounded for some continuance, its 12th and 17th are heard at the same time; and second, that if any two of the perfect accord of 3d, 5th, and 8th be sounded together, a third or fundamental sound will be heard. The causes of these phenomena have not hitherto been understood; they have, however, their origin in the complex motion of the strings. The present paper being already so far extended, I cannot here enter into the detail of the manner in which they are produced; but the fundamental cause of the wolf, as well as as the theory of harmony in general, I shall make the subject of a future communication.

DIVISION VIII. *Outline of an original System of the Physiology of the Ear.*

One part of the physiology of the ear has long been understood; namely, that the pinna collects the vibrations of the air, and transmits them through the meatus externus to the membrana tympani.

Another part of it, however, has not been understood; namely, that by it alone it is that we distinguish the direction of sounds. Ascending vibrations are distinguished by the coronal or upper part of the helix and concha; by the latter reflected directly basilad or downward, and by the former, along the fossa navicularis, to the meatus; descending vibrations are distinguished by the tragus, antitragus, and basilar or inferior part of the concha, and reflected from their obliquity in a direct line to the meatus; vibrations from before are distinguished by the inial or posterior part of the concha, whence they also are almost directly reflected; and vibrations from behind, impinging upon the tragus and antinial or fore part of the helix, are, by the former, carried directly, and by the latter, along the Fossa Navicularis, to the same parts.

It is no small confirmation of the preceding opinion, that the external ear is the sole organ by which we judge of the direction of sounds, that animals with cropped ears are generally less sensible of the direction of sound than those which possess them entire.

The vibrations thus received and distinguished, pass along the meatus externus, and by titilating its finely distributed nerves, which are branches of the Portio Dura, excite into action those other branches of the same nerve which supply the tympanic muscles. In consequence of this, these muscles instantly give that tension and direction to the Membrana Tympani, which is necessary for the reception of the impinging vibrations; and whatever actions are thus given to the Ossicula are communicated to the Chorda Tympani, a branch of the same nerve.

Now, to receive these vibrations, the Membrana Tympani is admirably adapted, being at all times slightly concave on its lateral or outer side, so that vibrations which tend in any particular direction, will readily glide from the other side, and impinge upon that side of the membrane which is most at right angles or opposed to their line line of percussion; and this I conceive to be the use of the various directions assumed by the membrane.

That it is by affecting the Meatus that the muscles are excited, I think much more probable than the hypothesis of Caldani, who supposes that these muscles are excited in consequence of some action on the Chorda Tympani. Now, before the Chorda Tympani could be affected, the vibrations must have already impinged upon the Membrana Tympani, and the subsequent action of the muscles upon it would be useless.

The membrane, then, having, as above described, assumed a proper degree of tension and inclination, the vibrations will pass in two directions across the Tympanum, namely, by the chain of bones, to the Membrana Fenestræ Ovalis, and, by the air of the Tympanum, to the Membrana Fenestræ Rotundæ, the inio-lateral or posterior and external aspect of which being, as remarked by Scarpa, concave, is, like the Membrana Tympani, adapted to receive the different directed vibrations.

Thus, we have arrived at the internal ear, a rational physiology of which is by far the most difficult. Previously, however, to attempting this, it is necessary again to observe, that sounds may exist in three different modes. They may be distinct, coexistent, or successive; and this indeed is the division which is made of them, however vaguely, in the present imperfect theory of music, where distinct sounds retain that name, coexistent sounds are termed Concords or Discords, and distinct or coexistent successive sounds are termed Melody or Harmony.

Now, in order to obtain a consistent physiology of the internal ear, it seems rational to look, in this complex organ, for three subordinate organs, namely, an organ for the impression of DISTINCT SOUNDS, an organ for the impression of COEXISTENT SOUNDS or concords and discords, and an organ for the impression of distinct or coexistent SUCCESSIVE SOUNDS or of melody and harmony.

Very curiously the Labyrinth or internal ear presents a corresponding arrangement into three parts, consisting of SEMICIRCULAR CANALS, COCHLEA, and VESTIBULUM; and, in these parts, we may rationally look for an organ of each of the modes of the existence of sound.

Now, as I have shown, that all sonorous vibrations and intervals must possess a semicircular arrangement, whatever their general direction may be; and as it is evident, that particular arrangements and particular directions of these vibrations are necessary to the existence of, or chiefly ly constitute particular sounds, it follows, that in order to make peculiar and corresponding impressions, these vibrations must, within the ear, pass in corresponding directions; and, for this purpose, an organ somewhat complex, consisting of channels in different directions, and these circular, must exist.

Having reached the vestibulum, it is therefore evident, that the vibrations will pass through and affect the nerves distributed in such a one of the semicircular canals as possesses the direction which corresponds with that peculiar movement of particles of which the vibration consists, and upon which the existence of the particular sound depends; and that, therefore, the SEMICIRCULAR CANALS CONSTITUTE THE ORGAN ON WHICH DISTINCT SOUNDS ARE IMPRESSED.

This Theory is admirably supported by the distribution of the branches of the Auditory Nerve upon the Membranous Semicircular Canals; for the sentient extremities of none of these nerves can be directly impressed from the Vestibulum, their sides only being turned to it; but as all of them enter the Semicircular Canals, the vibrations must pass in their particular direction, and through them before these nerves can be impressed.

Again, it is remarkable, that as there are only six simple directions, viz. upward, downward, backward, forward, to the right, and to the left, so the semicircular canals exactly permit these; for one of them is horizontal and two perpendicular; one of the perpendicular having its edge, the other its side turned forward. They do not, however, possess perfect regularity in this respect, and I shall, in a subsequent paper, show that the slight irregularity which takes place in their direction, and in the situation of their apertures within the Vestibule, is the cause of the most of those phenomena in music which have hitherto been unaccountable.

From all this, it follows, that by examining the circumference of these canals in any animal, we may easily ascertain what sounds it can distinguish. Too strong sounds will fall on the peripheral or external; too weak ones on the central or internal sides of the canals, and thus both will be rendered indistinct. In man, these canals are very equal in their circumference; and this is consistent with the rest of his structure. In birds and beasts of prey, which are calculated to distinguish the *minutest* sounds, some of them are, consistently with what I have just stated, ed, always of a proportionally *smaller* size, e. g. in the owl.

Not to be misled by the vibrations passing in different directions in the two ears, it is only necessary for a man to remember that he has two sides, and that what passes from right to left cannot pass from left to right. As to the prevention of mistakes respecting the nature of sound in two different persons from difference of situation, the external ear being, in all persons, analogously formed, will receive the analogous sounds, and lose those which are not so. The ear will thus distinguish, or rather be influenced by, the relations which, in any position, the directions of the vibrations bear to the vibrating body, and to the hearer. It is worth remarking, that the inial or posterior and coronal or superior situation of the pinna of the external ear, exactly corresponds to the situation of the semicircular canals of the internal. Thus, the pinna may be said to be the organ by which the difference of our position or of the direction of sound is corrected. — Here I shall at present leave the consideration of distinct sounds and their organ.

There must also exist a portion of the labyrinth, on which coexistent sounds may, at the same time, be impressed, or, in other words, an organ of Concords and Discords.

Now, the Cochlea very beautifully answers this purpose; for the air of the tympanum and the solid chain of bones will convey the vibrations in different times to its two Scalæ; and as these vibrations must be conveyed in different times to the Scalæ, it is evident that one series of them will reach the Tympanic Scala at the same moment that another series reaches the Vestibular. Hence two different series of vibrations must, at the same time, exist within the Cochlea, the one impressing the mesial or internal, the other the lateral or external side of the same nerves distributed upon the Zona Mollis, at the same instant. Thus, these nerves must be affected by the consonance or dissonance of the two coexistent series, and THE COCHLEA CONSTITUTES THE ORGAN OF CONCORDANT AND DISCORDANT SOUNDS.

It is some confirmation of this Theory, that singing birds have a larger Fenestra Rotunda and internal cavity than those which merely chirp or scream.

There must also exist a portion of the Labyrinth thro' which every sound, whether simple or complex, must pass in succession, and impress the same nerve so distributed, that that no vibration can escape it; in other words, an organ of Melody and Harmony.

As, therefore, all these vibrations must, in succession, pass through the Vestibule, and impress its branch of the Auditory nerve, which is so distributed upon the Sacculus Sphericus Vestibuli that no vibration entering the Labyrinth, in any direction, can escape it, that nerve must be impressed both by successive Distinct or Simple Sounds, and likewise by successive Concords or Discords, and THE VESTIBULE CONSTITUTES THE ORGAN OF MELODY AND HARMONY.

That the nerve of the Sacculus Sphericus radiates, and can be impressed in every direction, while those of the Canals can be impressed only in distinct directions, is no small confirmation of the preceding Theory.

## Figures and Tables

**Figure f1:**